# Post-surgical photobiomodulation therapy improves outcomes following elective gastropexy in dogs

**DOI:** 10.1007/s10103-024-04164-2

**Published:** 2024-08-08

**Authors:** J. C. Alves, Ana Filipe, Ana Santos

**Affiliations:** 1 Divisão de Medicina Veterinária, Guarda Nacional Republicana (GNR), Rua Presidente Arriaga 9, Lisbon, 1200-771 Portugal; 2 Faculty of Veterinary Medicine, Lusófona University, Lisbon, Portugal; 3 Centro de Ciência Animal e Veterinária, Lusófona University, Lisbon, 1749-024 Portugal; 4 MED – Mediterranean Institute for Agriculture, Environment and Development, Instituto de Investigação e Formação Avançada, Universidade de Évora, Pólo da Mitra, Ap. 94, Évora, 7006-554 Portugal; 5 Veterinary Teaching Hospital, Faculty of Veterinary Medicine, University of Lisbon, Lisbon, Portugal

**Keywords:** Photobiomodulation therapy, Dog, Pain, CMPS-SF, Pain scale

## Abstract

**Purpose:**

To evaluate the effect of post-surgical photobiomodulation therapy in dogs.

**Methods:**

Twenty dogs were selected for elective gastropexy and randomly divided into a control (CG, *n* = 10) and a PBMT group (PBMTG, *n* = 10). Pre‑medication consisted of medetomidine and butorphanol. Meloxicam was administered before the procedure. Induction was performed with propofol and maintained with sevoflurane. Local blocks with lidocaine were used. Incisional gastropexy was performed in all animals. PBMTG received PBMT immediately after surgery. The need for postoperative rescue analgesia, if the animal had eaten by the evaluation momen, and pain scores were collected using the Glasgow Composite Measure Pain Scale – Short Form (CMPS‑SF) at 1, 2, 4, 6, 8, 12, 16, 20, and 24 h post‑endotracheal extubation. CMPS‑SF scores were compared with the Mann-Whitney Test and proportions of animals that required rescue analgesia and had eaten with a χ^2^ test. P was set at < 0.05.

**Results:**

No rescue analgesia was needed for any animal. Still, significant differences were observed in CMPS-SF scores between CG and PBMTG between 1 and 4 h post-extubation. PBMTG had a significantly higher proportion of animals eating up to the 8 h post-extubation evaluation moment.

**Conclusion:**

Adding post-surgical photobiomodulation to a standard anesthesia and analgesia protocol reduced CMPS-SF scores and increased the proportion of animals that resumed eating compared to the standard protocol alone.

## Introduction

Gastric dilatation-volvulus (GDV) is commonly reported in large-breed, deep-chested dogs. Usually, it has an acute onset, making it a surgical emergency. Depending on the report, it has a high mortality, ranging from 9.8 to 28% in treated animals. If the cases where euthanasia is chosen instead of surgical treatment are added, the rate of nonsurvivors can reach 35.9% [1, 2]. Although the etiology is not fully understood, multiple risk factors have been identified, including breed (for example, Great Dane, Akita, Dogue de Bordeaux, German Shepherd Dog), increasing age, being neutered and male, increased thoracic depth-to-width ratio, being fed fewer meals per day or in a raised bowl, and exercise or stress around meals [3, 4]. The treatment of GDV includes encompasses medical stabilization followed by surgery. Surgery aims to reposition the stomach to its normal position and gastropexy. There are different techniques to perform gastropexy, but when proper technique is used, the risk of GDV is reduced to less than 5% [5]. For some animals, given the high risk of developing GDV, prophylactic gastropexy should be considered. Some agencies, employing a high number of animals at risk of developing GDV and following the death of many animals each year, elected to perform prophylactic gastropexy in all animals entering active service [6].

Several instruments are available to measure acute pain in dogs7,8, such as the Glasgow Composite Measure Pain Scale – Short Form (CMPS-SF). Its ability to evaluate post-surgical pain has been supported by different reports [7, 8]. The CMPS-SF includes six behavioral categories, with their associated descriptors: vocalization, attention to wound, mobility, response to touch, demeanor, and posture/activity [9]. This tool provides an important guide for veterinarians and reserach, since the final score guides to the need or not for additional analgesia [10]. Photobiomodulation therapy (PBMT), or low-level laser therapy, uses red/near-infrared light to produce a clinical effect. Different clinical effects have been described for PBMT, including the stimulation of tissue healing, analgesia, and a reduction in inflammation [11]. In dogs, PBMT has been described as managing osteoarthritis, chronic gingivostomatitis, diarrhea, wound healing, and improving outcomes following surgery [12–17].

This study aimed to evaluate the effect of post-surgical PBMT in dogs submitted to prophylactic gastropexy. We hypothesized that PBMT would contribute to lower post-surgical pain scores, decreasing the need for rescue analgesia.

## Materials and methods

Twenty dogs were recruited for the study from the Guarda Nacional Republicana (Portuguese Gendarmerie Canine Unit) police working dog population. No restrictions were placed on the age, breed, or sex of recruited dogs. The assisting veterinarian considered the animals at risk for GDV. A power analysis based on previous available data and comparison with the available literature, suggested that twenty animals would be sufficient to yield a power of 0.8 with an alpha value of 0.05. According to the applied intervention, the animals were randomly divided into a control group (CG, *n* = 10) and a PBMT group (PBMTG, *n* = 10). Group allocation was made following the random order determined with statistical analysis software.

### Surgical procedure

Standard anesthesia and analgesia were used for the surgical procedure. All dogs were pre‑medicated with an alpha2‑adrenoceptor agonist (medetomidine, 0.01 mg/kg) in combination with an opioid (butorphanol, 0.1 mg/kg), administered intravenously. Induction of anesthesia was performed with propofol (1-4 mg/kg) and maintained with sevoflurane. Meloxicam (0.2 mg/kg) was administered before the surgical procedure. Local anesthetic blocks, using lidocaine (4 mg/kg), were used in both groups and included incisional blocks in the skin and the abdominal wall. For post‑operative pain relief, buprenorphine (0.02 mg/kg) was available and used for rescue analgesia. The need for rescue analgesia was determined with the CMPS‑SF and set as a score ≥ 6 [18].

The surgical procedure followed a standard approach for incisional gastropexy, as described elsewhere [5]. Briefly, after assessing the abdominal cavity through laparotomy, an incision of 5 cm was performed in the seromuscular layer of the gastric wall, extending through the seromuscular. The incision was made at the level of the pyloric antrum, parallel to the long axis of the stomach, in an area in a few blood vessels between the lesser and greater curvatures. Caudal to the last right rib, midway from the ventral and dorsal limits of the abdominal cavity, an incision of equal length was made through the peritoneum and right transversus abdominis muscle, parallel to the muscle fiber direction. The two incisions were sutured together in two simple continuous pattern using a 2 − 0 monofilament suture of glycomer 631, composed of glycolide, dioxanone, and trimethylene carbonate. A retrospective study showed that incisional gastropexy had an equivalent result of belt-loop gastropexy in preventing GDV and higher efficacy than circumcostal gastropexy and gastrocolopexy [5]. The same researcher performed all surgical procedures. Animals were offered food once fully awake.

### Photobiomodulation therapy

PBMTG received PBMT immediately after surgery with a therapeutic laser (CTS-DUO Class IV Laser, Companion Animal Health, Enovis). A single session was performed, covering the entire abdominal area. PBMT parameters are presented in Table 1. The same researcher performed all PBMT treatments.

**Table 1:** Photobiomodulation therapy treatment parameters

Light Parameters (Dose)	
**Wavelength (nm)**	980 (for patients with dark coat color) 980/808 blend (for patients with light to medium coat color)
**Radiant Power (W)**	12
**Irradiance (W/cm2) at skin surface**	1,5
**Fluence (J/cm** ^**2**^ **)**	8.6 (average over treated area)
**Total Joules**	4,320
**Treatment Protocol**	Continuously moving grid pattern in contact over the area of the greater trochanter at a speed of 2.5–7.5 cm/second, according to manufacturer recommendations
**Treatment Area (cm** ^**2**^ **)**	500
**Treatment Time**	6 min

### Collected data

The Portuguese version of the CMPS‑SF was validated before this study (not reported here). All scoring procedures were performed by the same researcher, blinded to the animals’ assigned group. Demographic and surgical procedural details, the time of endotracheal extubation, the need for post‑operative rescue analgesia administration, and whether the animal had eaten by the evaluation moment were recorded for all dogs. Food was offered to the animals once they were fully awake. Pain scores were collected using the CMPS‑SF at 1, 2, 4, 6, 8, 12, 16, 20, and 24 h post‑endotracheal extubation.

### Statistical analysis

CMPS‑SF scores were compared at each evaluation moment with the Mann-Whitney Test. The proportion of animals in each group that required rescue analgesia that had eaten was submitted to a χ^2^ test with Yates correction in each evaluation moment. The effect size was determined, and considered to be small if *r* < 0.3, medium if 0.3 < *r* < 0.5, and large if *r* > 0.5. All results were analyzed using commercially available software (IBM SPSS Statistics version 20). Significance was set at *p* < 0.05.

## Results

The sample included 20 dogs, with a mean body weight of 24.1 ± 5.1 kg and a mean age of 3.4 ± 2.1 years. Both sexes were represented, with 11 females and 9 males, and three breeds were represented: Belgian Malinois Shepherd Dogs (*n* = 13), German Shepherd Dogs (*n* = 5), and Dutch Shepherd Dogs (*n* = 1).

**Fig. 1:**
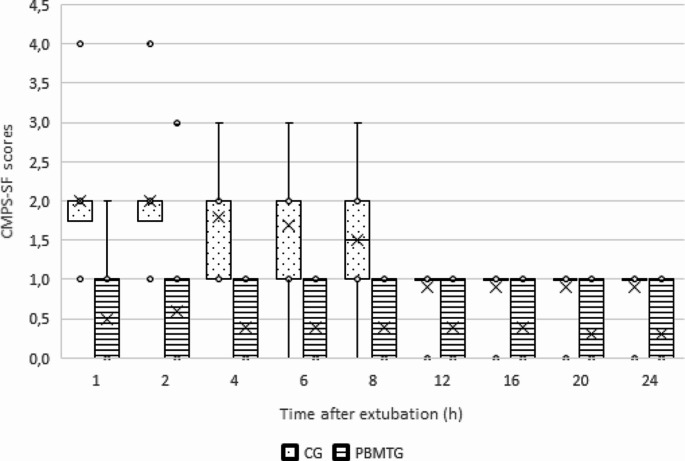
Glasgow Composite Measure Pain Scale – Short Form (CMPS-SF) scores in the control group (CG) and photobiomodulation therapy group (PBMTG). A significant difference was observed between the two groups up to the 8 h evaluation moment. Box plots represent the median. 25th. and 75th percentiles. and whiskers represent the 10th and 90th percentiles

Even though no rescue analgesia was needed for any animal, indicating that the established anesthesia and analgesia protocol effectively managed pain, significant differences were observed in CMPS-SF scores between CG and PBMTG between 1 and 4 h post-extubation. Animals in PBMTG also started to eat soon, with a significantly higher proportion of animals having eaten up to the 8 h post-extubation evaluation moment, which may indicate a higher level of comfort. A large effect size was also observed in these evaluation moments. The results obtained for each group of dogs with the CMPS-SF are the proportion of animals that had eaten and received rescue analgesia, are presented in Table 2. The evolution of CMPS-SF scores is shown in Fig. 1.

**Table 2:** Results (median and interquartile range) of Glasgow Composite measure Pain Scale – Short Form. Percentage of animals that had eaten and received rescue analgesia, and effect size in control (CG) and photobiomodulation therapy (PBMTG) groups

**Group**	**+ 1 h**										**+ 2 h**										**+ 4 h**									
	**score**	**IQR**	**p**	**ES**	**Analgesia**	**p**	**ES**	**Eaten**	**p**	**ES**	**score**	**IQR**	**p**	**ES**	**Analgesia**	**p**	**ES**	**Eaten**	**p**	**ES**	**score**	**IQR**	**p**	**ES**	**Analgesia**	**p**	**ES**	**Eaten**	**p**	**ES**
**CG**	2,0	0,0	< 0,01	0,95	0%	1,00	0,03	50%	0,01	0,82	2,0	0,0	< 0,01	0,80	0%	1,00	0,10	60%	0,02	0,89	2,0	0,8	< 0,01	0,95	0%	1,00	0,10	80%	0,03	0,71
**PBMTG**	0,0	1,0			0%			100%			0,0	1,0			0%			100%			0,0	1,0			0%			100%		
**Group**	**+ 6 h**										**+ 8 h**										**+ 12 h**									
	**score**	**IQR**	**p**	**ES**	**Analgesia**	**p**	**ES**	**Eaten**	**p**	**ES**	**score**	**IQR**	**p**	**ES**	**Analgesia**	**p**	**ES**	**Eaten**	**p**	**ES**	**score**	**IQR**	**p**	**ES**	**Analgesia**	**p**	**ES**	**Eaten**	**p**	**ES**
**CG**	2,0	0,8	0,48	0,00	0%	1,00	0,47	80%	0,03	0,67	1,5	1,0	0,13	0,14	0%	1,00	0,47	80%	0,03	0,57	1,0	0,0	1,00	0,03	0%	0,14	0,15	100%	1,00	0,20
**PBMTG**	0,0	1,0			0%			100%			0,0	1,0			0%			100%			0,0	1,0			0%			100%		
**Group**	**+ 16 h**										**+ 20 h**										**+ 24 h**									
	**score**	**IQR**	**p**	**ES**	**Analgesia**	**p**	**ES**	**Eaten**	**p**	**ES**	**score**	**IQR**	**p**	**ES**	**Analgesia**	**p**	**ES**	**Eaten**	**p**	**ES**	**score**	**IQR**	**p**	**ES**	**Analgesia**	**p**	**ES**	**Eaten**	**p**	**ES**
**CG**	1,0	0,0	1,00	0,00	0%	0,36	0,10	100%	1,00	0,10	1,0	0,0	0,50	0,00	0%	1,00	0,10	100%	0,14	0,10	1,0	0,0	0,50	0,00	0%	1,00	0,10	100%	0,63	0,10
**PBMTG**	0,0	1,0			0%			100%			0,0	0,8			0%			100%			0,0	0,8			0%			100%		

## Discussion

In the present study, adding post-surgical photobiomodulation to a standard anesthesia and analgesia protocol reduced CMPS-SF scores and increased the proportion of animals that resumed eating compared to the standard protocol alone.

The use of a multimodal approach, based on non-steroidal anti-inflammatory drugs (NSAIDs) and opioids, is commonly considered the gold standard of postoperative analgesia [19]. In different instances, and for multiple reasons, alternatives to opioids may be considered, and include the use of local anesthetics. This approach is frequently referred to as opioid-sparing or opioid-free anesthesia. A regional technique can often reduce the dose of other anesthetic drugs required to maintain anesthesia and analgesia following the procedure technique [18]. The standard protocol used in the present study adequately managed pain in dogs submitted to prophylactic gastropexy, as the documented threshold for the need for rescue analgesia was never reached in any animal. This finding is reinforced by a single researcher’s frequent and standardized scoring of postoperative pain, further supporting multimodal analgesia protocols. Post-operative pain can also be influenced by the surgeon’s experience, as an inexperienced surgeon may induce higher levels of inflammation due to higher surgical time and tissue handling technique [20]. Surgical site inflammation, although usually self-limiting, can generate physical discomfort [21]. In the present study, all procedures were performed by the same experienced surgeon, which reduces variability between groups and reduces the limitations of a less inexperienced surgeon.

The effects of PBMT on inflammation and pain, particularly chronic pain, are well documented [11, 16, 17, 22]. These may be the reasons for the difference in results observed between the two groups. Even though any animal in the study required rescue analgesia, significantly lower pain levels were observed in PBMTG up to the 6 h evaluation moment. As shown in Figure, CMPS-SF scores were also more homogenously low, with fewer outliers. Although it was not routinely recorded for the study, several handlers of dogs in PBMTG reported that, where led outside to go to the bathroom, many dogs would lie on their backs, comfortably spread, which was interpreted as a sign of comfort. A similar effect during/following abdominal PBMT has been described before [16]. Different mechanisms have for the analgesic effects of PBMT, inluding an increased in the secretion of endorphin in inflammation sites, allied to an enhanced circulation. In addition, PBMT can aid in the release of neurotransmitters, decrease the production of prostaglandin E_2_, cyclooxygenase-2 and nociceptor signal transduction [23].

Another sign of comfort showed by animals in both groups, but in a higher proportion in PBMTG up to the 8 h evaluation moment, was the fact that animals accepted food early on. In fact, all animals in PBMTG accepted food before the first evaluation moment. PBMT can also help manage surgical site inflammation since it is performed immediately after surgical inflammation is approached before it has the chance to settle in. This should be assessed in future studies and incidence of other minor complications, such as seroma formation. PBMT has been shown to modulate the expression of inflammatory mediators, including interleukins 1, 6, 10 and tumor necrosis factor-alpha, in addition to some antimicrobial properties that can help to reduce the inflammatory process [24].

This study has some limitations. One is related to pain assessment accuracy, a limitation inherent to all approaches to measuring pain in animals. Still, the CMPS-SF has been extensively evaluated, and composite pain scales, as a whole, are more sensitive and specific than other methods [21]. An additional limitation concerns the nature of the animals included in the sample. Working dogs are commonly considered stoic dogs, tending to hide or not exhibit overt signs of pain [25–27]. Still, the researcher who carried out the evaluations has vast experience with these animals and is familiar with their normal behavior. A final limitation is related to the fact that a single procedure was considered in this study. Future studies should evaluate the inclusion of PBMT in different procedures.

## Conclusion

The inclusion of a single session of post-surgical PBMT reduced pain scores in animals submitted to elective gastropexy compared to a standard analgesia and anesthesia protocol. Nevertheless, the CMPS-SF scores remained below the threshold for rescue analgesia in both groups. In addition to lower pain scores, PBMTG had a higher proportion of animals resuming eating from an earlier evaluation moment.
